# Enhancing result reliability by addressing potential confounding factors

**DOI:** 10.1128/spectrum.01327-24

**Published:** 2025-02-06

**Authors:** Satoru Mitsuboshi

**Affiliations:** 1 Department of Pharmacy, Kaetsu Hospital 38077 , Niigata, Japan; University of Pretoria, Pretoria, Gauteng, South Africa

**Keywords:** acute kidney injury, vancomycin, area under the concentration–time curve

## LETTER

I read Ishigo et al.’s Research Article with great interest (1). This study evaluated the relationship between area under the concentration–time curve (AUC) on day 2 of vancomycin and the incidence risk of acute kidney injury (AKI) in a Japanese intensive care unit setting. The results demonstrated that patients with AUCs 500 to 600 µg·h/mL were associated with an increase in the risk of AKI compared to patients with AUCs < 500 µg·h/mL. These findings suggest that the risk of AKI may vary even within the recommended AUC range for vancomycin as per current guidelines (2), potentially impacting future guideline recommendations. Although the study was elegantly designed, limited covariates were adjusted by Cox regression analysis, and this method is generally not considered a robust analysis (3). Therefore, I would suggest two additional analyses to address potential concerns.

First, I would suggest using a competing risk model where the possible endpoints are either AKI or death. This cohort included patients admitted to the intensive care unit and may have had a high mortality rate, although these data were not presented in the manuscript. Patients who died already cannot then have AKI, and the results may be misleading due to the competing risk of death. The Kaplan–Meier curves presented by the authors indicate that about 30% of patients had an observation period of 10 days or more. Namely, the incidence of AKI may be underestimated in groups with a high number of deaths.

Second, I would suggest using a doubly robust method that accounts for the inverse probability of treatment weighting at baseline and also adjusts for covariates in patients between the low-AUC group and intermediate-AUC group. Patients with higher AUCs may have received stronger treatments due to severe conditions, resulting in a higher risk of AKI. The study included a limited number of covariates adjusted by the Cox proportional hazards model. Thus, there may be many significant unadjusted confounding factors in this study, and more robust analyses may improve the reliability of this study.
